# Less, but not gone—gluten-free diet effects on fatigue in celiac disease: a prospective controlled study

**DOI:** 10.3389/fmed.2023.1242512

**Published:** 2023-09-12

**Authors:** Berit Mære Skjellerudsveen, Roald Omdal, Anne Kristine Hetta, Jan Terje Kvaløy, Lars Aabakken, Inger Marie Skoie, Tore Grimstad

**Affiliations:** ^1^Department of Internal Medicine, Stavanger University Hospital, Stavanger, Norway; ^2^Department of Clinical Science, University of Bergen, Bergen, Norway; ^3^Department of Mathematics and Physics, University of Stavanger, Stavanger, Norway; ^4^Department of Research, Stavanger University Hospital, Stavanger, Norway; ^5^Department of Transplantation Medicine, Rikshospitalet, Oslo University Hospital, Oslo, Norway; ^6^Department of Dermatology, Stavanger University Hospital, Stavanger, Norway

**Keywords:** celiac disease, fatigue, quality of life, immunology, gastroenterology

## Abstract

**Introduction:**

Fatigue is a frequent complaint in patients with celiac disease. A gluten-free diet is the only established treatment for celiac disease, but how this diet influences fatigue is uncertain. We aimed to investigate fatigue prevalence, severity, and associated factors in patients with celiac disease, at diagnosis and at 1 year after commencing a gluten-free diet.

**Methods:**

78 patients with serologically and histologically verified celiac disease, 78 age- and sex-matched healthy subjects. Primary endpoints were Fatigue Visual Analog Scale (fVAS), Fatigue Severity Scale (FSS), and inverted Vitality subscale of the Medical Outcomes Study 36-Item Short-Form Health Survey (SF-36vs). Clinically relevant fatigue was defined as: FSS score ≥ 4, fVAS score ≥ 50 mm, or inverted SF-36vs score ≥ 65. Higher scores represented more fatigue.

**Results:**

Fatigue was reduced after a 12-month gluten-free diet. Median scores changed from 3.8 (interquartile range [IQR]: 2.2 to 4.8) to 1.9 (IQR: 1.4 to 3.5) for FSS, from 44.5 (IQR: 18.8 to 66.0) to 15.5 (IQR: 7.8 to 43.3) for fVAS, and from 65 (IQR: 40 to 75) to 35 (IQR: 25 to 55) for inverted SF-36vs (*p* < 0.001 for all). Fatigue prevalence also declined after treatment. However, scores were significantly higher in patients compared to control subjects. Higher fatigue scores were associated with depression and pain, but not with signs of disease activity or nutritional deficiency.

**Conclusion:**

At diagnosis, patients with celiac disease frequently had severe fatigue. Fatigue declined after a gluten-free diet, but it remained higher than that observed in healthy subjects.

**Clinical trial registration:**

ClinicalTrials.gov, Identifier NCT01551563.

## Background

Celiac disease (CD) is a chronic, immune-mediated disease that develops in genetically predisposed individuals. CD is triggered by the ingestion of gluten, and has been increasingly recognized in adults. The worldwide prevalence is about 1.4% (1, 2). The small intestine is the primary organ affected. The gluten-driven immune response causes mucosal injury, which leads to malabsorption, abdominal pain, and diarrhea. However, the clinical manifestations are broad, with diverse intestinal and extra-intestinal symptoms and signs. Fatigue, headaches, and joint pain are frequent complaints, and often, they occur without an awareness of intestinal symptoms. The disease mechanisms underlying these extra-intestinal phenomena are incompletely understood (3).

Although new treatment options for CD are currently under investigation, a strict gluten-free diet (GFD) remains the only effective treatment option (4, 5). This mandatory restrictive diet may affect quality of life negatively; as much as 40% of patients report difficulties in adequately adhering to the GFD (6, 7).

Fatigue, often defined as “an overwhelming sense of tiredness, lack of energy, and feeling of exhaustion” (8) is a frequent phenomenon in all chronic inflammatory, neurodegenerative, and malignant diseases (9, 10). It can be conceptually understood as a major component of the “sickness behavior response.” This response is a complex, evolutionarily conserved survival mechanism observed in animals and humans during states of infection and injury (11). Sickness behavior is characterized by fatigue, depression, social withdrawal, and reduced thirst and appetite (11). The genetic background and mechanisms for this response are complex, but a number of animal and human studies have highlighted the central roles of signaling molecules, like IL-1β, which induce neuronal activation in the brain (12, 13). Fatigue occurs with the activation of innate and adaptive immune responses, and it is modulated by psychosocial factors, like pain and mood.

Fatigue is a frequent cause for sick leave, and many patients report fatigue as their worst problem. There is no gold standard for evaluating or grading fatigue. Moreover, it is debated whether there are different dimensions of fatigue, like muscular, mental, and cognitive fatigue, or whether fatigue is a unidimensional condition that influences different dimensions or aspects of life. This conundrum is reflected in the multitude of different instruments available for measuring fatigue. Some instruments are generic, and others are disease-specific. These inconsistencies and the lack of a widely accepted definition of fatigue have made it difficult to compare results from different studies and to obtain a universal understanding of fatigue.

Previous studies of fatigue in CD have been hampered by these limitations. A recent review illustrated the problem with reported frequencies of fatigue in CD ranging from 8 to 100% (14). Nevertheless, fatigue seems to represent a major problem in CD; it leads to considerably lower quality of life, and it is often accompanied by depression and pain (15, 16). Notably, few studies have investigated how a GFD influences fatigue in CD, and the available conclusions have been inconsistent: some studies reported that a GFD provided improvement, and others found no change in fatigue severity (16–19).

In this one-year observational study, we aimed to investigate the course of fatigue, with emphasis on fatigue prevalence and severity in patients with CD, from the time of diagnosis to 1 year after commencing a GFD. We measured fatigue with validated and widely accepted generic, unidimensional instruments.

## Materials and methods

### Patient recruitment and diagnostic procedures

This single-center, prospective, controlled study was performed at the Unit of Gastroenterology, Department of Internal Medicine, Stavanger University Hospital, Norway. Patients referred with clinically suspected CD were eligible for inclusion, when they had elevated anti-tissue transglutaminase-IgA antibodies (anti-tTG-IgA) at the time of referral (≥7 U/mL), were aged ≥ 18 years, and consumed a gluten-containing diet. The final inclusion criterion was: histopathological findings consistent with CD. Exclusion criteria were an inability to consent and non-adherence to the treatment protocol.

At the first study visit (V0), an upper endoscopy was performed. Six mucosal biopsies were obtained: four from the descending part of the duodenum and two from the proximal duodenal bulb.

### Patient handling

Eligible patients were informed about the study, provided written informed consent before inclusion, and were invited to a follow-up visit at 12 months after GFD initiation (V12). At the V0 and V12 study visits, demographics and clinical data were recorded, and blood samples were analyzed. All patients underwent a gastroscopy at V0. At V12, a second gastroscopy was scheduled, but not mandatory. Data were electronically recorded on an iPad case report form with FileMaker Pro software (Claris International).

### Introduction of a gluten-free diet

All patients were invited to a one-day course at the “Learning and coping center” at Stavanger University Hospital shortly after confirming the diagnosis. A licensed nutritionist provided comprehensive information about the GFD and how to adhere to this diet. Patients that did not attend this course (*n* = 7) were contacted by a study nurse to confirm their compliance to the diet. Participants had to confirm diet adherence throughout the study period at the V12 visit.

### Healthy subjects and matching procedure

Seventy-eight self-reported healthy control subjects were included in the study. The matching criteria were sex and age ± 5 years. The majority was recruited from non-family acquaintances of patients, and approximately 1/3 were recruited from employees of the hospital and their acquaintances. Measures of fatigue, mood, and quality of life were recorded only once. No other clinical data on the control subjects were recorded.

### Demographic and clinical data

Age, sex, and body mass index (BMI) were recorded for all study participants. Concurrent autoimmune diseases were recorded, based on patient self-reporting and medical files.

### Blood tests

At V0 and V12, hemoglobin, folic acid, cobalamin, 25-hydroxy vitamin D, anti-tTG-IgA, and anti-deamidated gliadin peptide IgG antibodies (DGP-AGA IgG) were analyzed in routine hospital laboratory tests. Ferritin levels were recorded from measurements performed within 3 months of V0 and at V12.

### Histopathological evaluation

Duodenal biopsies obtained at V0 and V12 were examined at the hospital’s Department of Pathology. All biopsies were formalin-fixed, paraffin-embedded, stained with hematoxylin and eosin, and graded according to the modified Marsh-Oberhuber classification (20).

### Fatigue assessment

Fatigue severity was graded with three generic, unidimensional fatigue instruments: the fatigue Visual Analog Scale (fVAS); the Fatigue Severity Scale (FSS), and the Vitality subscale of the Medical Outcomes Study 36-Item Short-Form Health Survey (SF-36vs) (21–23). These questionnaires were completed on site by patients at V0 and V12 under the instruction and supervision of a study nurse.

The fVAS comprises a 100-mm horizontal line with vertical anchors; the left end of the line (0 mm) is labeled “no fatigue,” and the right end (100 mm) is labeled “fatigue as bad as it can be.” The participant rated the severity of fatigue in the last week by drawing a vertical line between the two ends, and the distance in millimeters was the fatigue score (22).

The FSS contains nine statements regarding fatigue. Responses were rated on a scale of 1 to 7, where higher numbers indicated more fatigue. The FSS score was the mean score of the nine questions, and it ranged from 1 to 7 (23).

The SF-36vs contains four items that address energy and fatigue, yielding combined scores between 0 and 100, where a higher score indicated higher vitality (21). For this study, we inverted the recorded SF-36vs scores to facilitate comparisons with the other scales and for illustrative purposes. Thus, we defined fatigue as follows: inverted SF-36vs = 100 − SF36vs.

Clinically relevant or significant fatigue was defined as follows: fVAS ≥ 50; FSS ≥ 4; and SF-36vs ≤ 35 or inverted SF-36vs ≥ 65 (24–27).

### Depression and pain

Depression was evaluated with the Hospital Anxiety and Depression Scale, Depression Subscale (HADS-D). Higher scores indicated more depression (28).

Pain was rated with the Medical Outcomes Study 36-Item Short Form Health Survey (SF-36) questionnaire, bodily pain subscale (SF-36 pain) (21). The SF-36 pain subscale consists of two items, one rates body pain intensity and the other rates the degree to which pain interferes with normal activities. The combined scores ranged from 0 to 100, and higher scores indicated less pain. Again, we inverted the pain scores (i.e., inverted score = 100 − base score) to facilitate comparisons with the other scales.

### Quality of life measures

Quality of life was assessed with the SF-36 questionnaire (21). The scores ranged from 0 to 100, and higher scores indicated better quality of life.

### Statistical analysis

We determined the sample size for this study to ensure sufficient power for detecting clinically relevant differences in fatigue scores. By including 78 patients with CD, we estimated 90% power for detecting a difference in fVAS scores of at least 9 points, assuming a standard deviation of 23 points, as previously reported (18). Previously, a difference of 20 points in fVAS scores was regarded clinically relevant (29, 30).

For continuous data, we performed the Shapiro–Wilk test to test for normal distributions. We conducted pairwise comparisons between patients at V0 and V12 and between patients at V12 and their individually matched healthy subjects. For those comparisons, we analyzed normally distributed data with the paired samples t-test, and we analyzed non-normally distributed data with the Wilcoxon paired samples rank test. For categorical variables, we performed the McNemar test. Confidence intervals for median changes were calculated with bootstrapping. For comparisons between independent groups, we performed the Mann–Whitney and Chi-Squared tests, as appropriate.

We performed univariable linear regression analysis with the fVAS, FSS, and inverted SF-36vs as dependent variables. Associations were examined with the following independent variables: sex, age, BMI, hemoglobin, folic acid, cobalamin, 25-hydroxy vitamin D, anti-tTG-IgA antibodies, ferritin, Marsh-score, HADS-D, and inverted SF-36 pain.

Independent variables in the univariable regression analyses that showed *p*-values < 0.2 were used to develop multivariable regression models. Backward and forward selections were performed to establish a final multivariable regression model. Analyses were performed with IBM SPSS statistical software (Version 26.0. IBM Corp.). *p*-values < 0.05 were considered statistically significant. For all regression models, we report standardized beta coefficients, which are interpreted as the impact of a one-standard-deviation increase in the corresponding independent variable.

### Ethical considerations

This study was approved by the Regional Ethics Committee (REC South-East Norway 2011/2631). It was conducted in compliance with the principles of the Declaration of Helsinki. The study was registered at ClinicalTrials.gov (NCT01551563).

## Results

**Figure 1:**
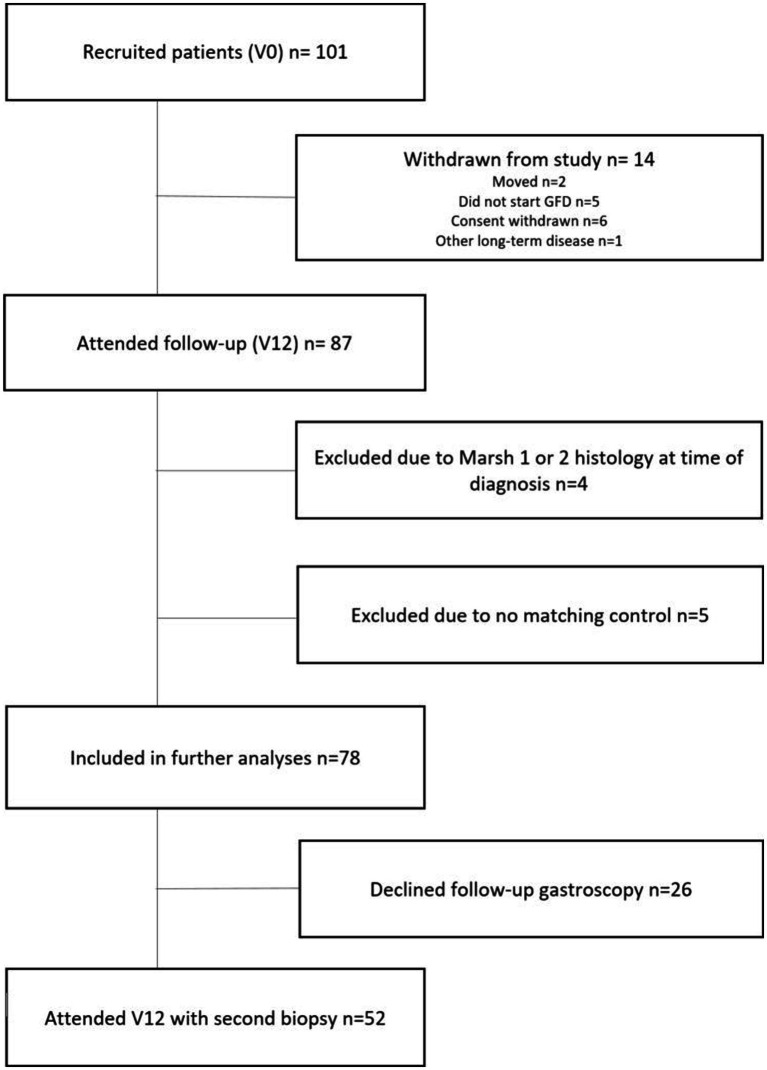
Flowchart shows the selection of patients with celiac disease for study inclusion and analyses. Inclusion period: 1 December 2016–30 September 2018. Follow-up period: 18 January 2018–16 September 2020; V0, Baseline; V12, Follow-up after 12 months of a gluten-free diet.

During the inclusion period, between 1 December 2016 and 30 September 2018, 125 subjects were screened for participation. A diagnosis of CD was established in 101 cases (80.8%). Fourteen patients withdrew, and nine were excluded. Thus, 78 patients and matching controls were included for further study (Figure 1, flowchart).

### Demographics and clinical data

Characteristics of the 78 study participants are given in Table 1. The median age at diagnosis was 40 years, and 45 patients (58%) were females.

**Table 1:** Demographics and laboratory findings at diagnosis and after 1 year of consuming a gluten-free diet in 78 patients with celiac disease.

Variables^*^	Patients at V0	Patients at V12	Change from V0 to V12 (95% CI)	value of *p* for V0–V12 difference	Healthy control subjects
Age (years)	40 (18–76)	41 (19–78)			40 (19–71)
Male	33 (42%)	33 (42%)			33 (42%)
Female	45 (58%)	45 (58%)			45 (58%)
BMI (kg/m^2^)	24.1 (23.2–24.9)	24.2 (23.3–25.1)	0.02 (−0.2–0.3)	0.304	25.2 (24.2–26.1)
Cobalamin (pmol/L)	369 (145–1,400)	338 (177–1,400)	19.0 (−1.0–29.0)	0.19	
Ferritin (μg/L)	53 (6–443)	81 (9–289)	17.5 (10.5–27.5)	<0.001	
Folic acid (nmol/L)	11.5 (3.4–39.0)	16.0 (2.4–62.9)	3.4 (3.0–5.6)	<0.001	
Hemoglobin (g/dL)	14.0 (13.7–14.3)	13.9 (13.6–14.2)	0.1 (−0.3–0.1)	0.371	
25-Hydroxy vitamin D (nmol/L)	67.7 (62.8–72.5)	74.0 (69.0–79)	6.74 (2.1–11.4)	0.005	
Anti-tTG-IgA (U/mL)	53 (5–141)	4.1 (0.4–82)	−40.3 (−68.0−−31.5)	<0.001	
DGP-AGA (U/mL)	24.0 (0.9–301.0)	2.4 (0.4–54.0)	−20.6 (−29.0−−9.3)	<0.001	
Marsh classification				<0.001	
0	0	33 (42%)			
1	0	7 (9%)			
2	0	0			
3a	15 (19%)	10 (13%)			
3b	29 (37%)	1 (1%)			
3c	34 (44%)	1 (1%)			

Data are the median (range), except BMI, hemoglobin, and 25-hydroxy vitamin D, which are the mean (SD), and sex, Marsh classification, which are the absolute number (%). Changes from V0 to V12 are presented as the median (95% CI), except for the changes in hemoglobin and 25-hydroxy vitamin D, which are presented as the mean (95% CI). ^*^Some values were missing, as follows: in the V0 group: *n* = 1 missing for hemoglobin and *n* = 7 missing for ferritin; in the V12 group: *n* = 26 missing for Marsh-scores (due to no endoscopy), *n* = 3 missing for ferritin, *n* = 2 missing for hemoglobin, *n* = 7 missing for tTG-IgA, *n* = 5 missing for cobalamin, *n* = 5 missing for folic acid, and *n* = 2 missing for 25-hydroxy vitamin D. BMI, Body mass index; CI, Confidence interval; DGP-AGA, Anti-deamidated gliadin peptide IgG antibodies; Anti-tTG-IgA, Anti-tissue transglutaminase IgA antibodies; V0, First study visit; V12, Follow-up visit after 12 months of a gluten-free diet.

After 12 months of GFD, participants showed increases in ferritin, folic acid, and 25-hydroxy vitamin D levels. Anti-tTG-IgA antibody levels declined. In 44 patients (62%), the V12 anti-tTG-IgA antibody was in the normal range, but in 27 patients (38%), the V12 levels remained above the normal range. DGP-AGA IgG levels also declined. The Marsh score declined; villous atrophy (Marsh 3a-c) was detected in all 78 patients at diagnosis, but in only 12 patients (23% of those that underwent a second gastroscopy) at V12.

Sixteen patients (21%) had one or more concomitant autoimmune disease at V12. These diseases included: diabetes mellitus type 1 (*n* = 3), dermatitis herpetiformis (*n* = 2), primary biliary cholangitis (*n* = 1), psoriasis (*n* = 4), rheumatoid arthritis (*n* = 1), and thyroid disease (*n* = 6). One patient with thyroid disease had Graves’ disease; the others had hypothyroidism treated with thyroid hormone.

The median observation time from V0 to V12 was 14 months. One patient attended the follow-up visit at 32 months after V0, due to late GFD initiation, to ensure 12 months on the GFD before the follow-up analysis. One patient attended V12 after 11 months and 3 weeks. All other patients attended V12 at 12 to 18 months after V0.

### Fatigue measures

#### Fatigue severity from baseline to follow-up (V0 vs. V12)

**Figure 2:**
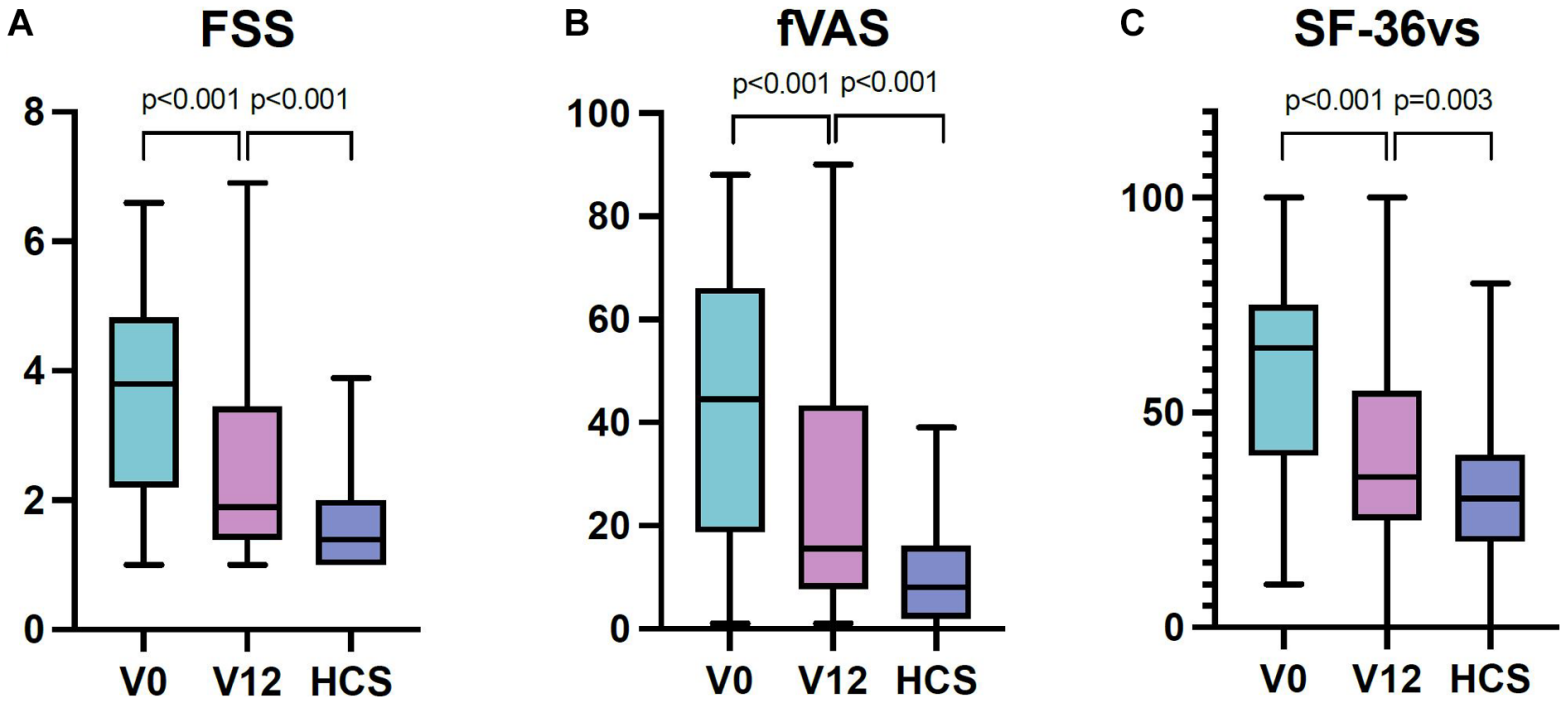
**(A-C)** FSS, fVAS, and (inverted) SF-36 vitality scores in patients with celiac disease at diagnosis (V0), after 1 year of a gluten-free diet (V12), and in healthy control subjects. Lines indicate medians. Boxes indicate interquartile ranges. Whiskers indicate ranges. FSS, Fatigue Severity Scale; fVAS, fatigue Visual Analog Scale; SF36vs, Vitality subscale of the Medical Outcomes Study 36-Item Short-Form Health Survey; HCS, Healthy control subjects; SF36vs scores were converted to reflect fatigue, as follows: inverted SF-36vs = 100 − SF-36vs.

In all three instruments, fatigue scores were significantly reduced at V12. FSS scores dropped from a median of 3.8 (interquartile range [IQR]: 2.2 to 4.8) to 1.9 (IQR: 1.4 to 3.5); fVAS scores dropped from 44.5 (IQR: 18.8 to 66.0) to 15.5 (IQR: 7.8 to 43.3); and inverted SF-36vs scores dropped from 65 (IQR: 40 to 75) to 35 (IQR: 25 to 55). *p*-values were <0.001 for all three differences (Figures 2A–C). The mean changes in fatigue scores from V0 to V12 were: FSS: −1.06 (95% CI: −1.43 to −0.68), fVAS: −16.0 (95% CI: −22.4 to −9.9), and inverted SF-36vs: −19.0 (95% CI: −24.1 to −14.0).

#### Fatigue severity at follow-up vs. healthy control subjects

In all three instruments, the fatigue scores were higher in patients after the GFD compared to healthy control subjects. The median FSS scores were 1.9 (IQR: 1.4 to 3.5) vs. 1.4 (IQR: 1.0 to 2.0, *p* < 0.001); the fVAS scores were 15.5 (IQR: 7.8 to 43.3) vs. 8.0 (IQR: 2.0 to 16.0, *p* < 0.001); and the inverted SF-36vs scores were 35 (IQR: 25 to 55) vs. 30 (IQR: 20 to 40, *p* = 0.003), respectively (Figures 2A–C).

#### Fatigue prevalences

**Figure 3:**
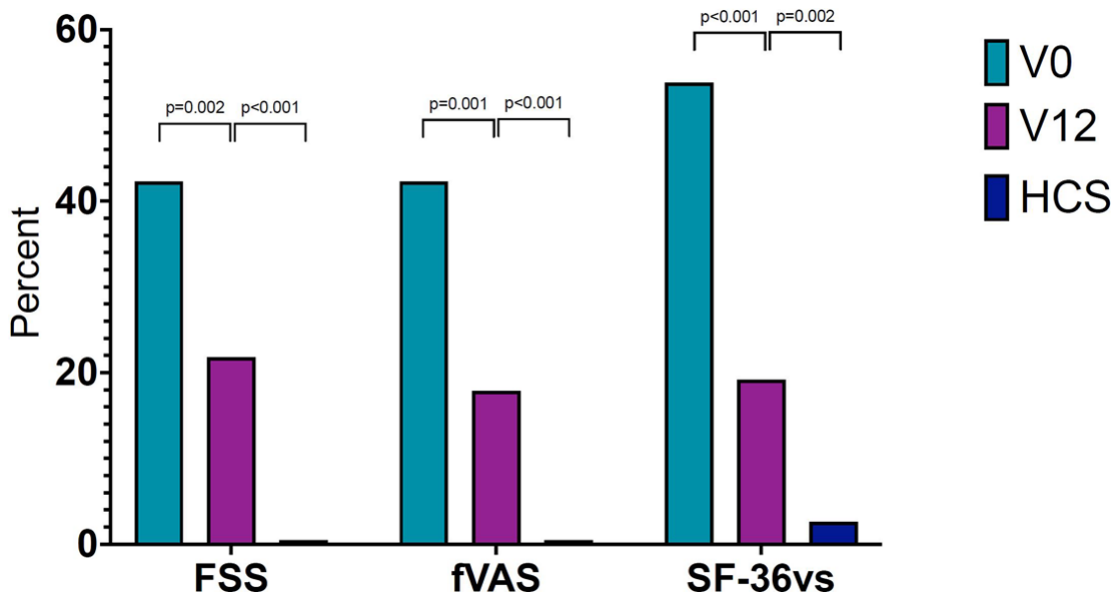
Prevalence of clinically relevant fatigue in patients with celiac disease. Fatigue prevalences in 78 patients with celiac disease, at diagnosis (V0) and after 1 year of a gluten-free diet (V12). Prevalences in healthy control subjects are shown for comparison. Fatigue was evaluated with three different instruments. FSS, Fatigue Severity Scale; fVAS, fatigue Visual Analog Scale; SF36vs, Vitality subscale of the Medical Outcomes Study 36-Item Short-Form Health Survey; HCS, Healthy control subjects; SF36vs scores were converted to reflect fatigue, as follows: inverted SF-36vs = 100 − SF-36vs.

The prevalence of clinically relevant fatigue measured at V0 was reduced after 1 year of GFD (V12). Prevalences fell from 42 to 22%, based on the FSS (*p* = 0.002), from 42% (V0) to 18% (V12), based on the fVAS (*p* = 0.001), and from 54% (V0) to 19% (V12), based on the SF-36vs (*p*< 0.001). In healthy control subjects, the prevalence rates were 0% (FSS), 0% (fVAS), and 3% (SF-36vs; Figure 3).

### Factors associated with fatigue after 12 months of a gluten-free diet

#### Univariable regression analysis

Depression and pain scores were positively associated with scores from all three measures of fatigue. Ferritin was negatively associated with all three scores. Folic acid showed a positive association with fVAS and FSS scores. Female sex was only associated with inverted SF-36vs scores (Supplementary Table S1).

We found no significant associations between any of the fatigue scores and BMI, cobalamin, DGP-AGA IgG, hemoglobin, Marsh-classification, anti-tTG-IgA, or 25-hydroxy-vitamine D (Supplementary Table S1).

#### Multivariable analysis

In the multivariable regression analysis, the HADS-D and SF-36 pain scores showed the strongest contributions to fatigue measured with the FSS, fVAS, or inverted SF-36vs (evaluated in three separate models). In addition, female sex was included in the final inverted SF-36vs model, and folic acid was included in the final fVAS model (Table 2). Applying forward and backward selections resulted in the same model results. The FSS, fVAS, and inverted SF-36vs models explained 53, 59, and 53% of the variability, respectively (Table 2).

**Table 2:** Factors that significantly influenced fatigue in 78 patients with celiac disease after 1 year of a gluten-free diet.

Variables	FSS		fVAS		SF-36vs (inverted)	
	β	value of *p*	β	value of *p*	β	value of *p*
Folic acid			0.201	0.013		
HADS-D (depression)	0.518	<0.001	0.568	<0.001	0.561	<0.001
Sex					−0.244	0.004
SF-36 pain (inverted)	0.348	0.003	0.256	0.003	0.237	0.01
R^2^ for multiple regression model	0.527		0.585		0.526	

Results are from the final multiple regression analysis model, performed with forward and backward model selection. The results were the same, when *p* < 0.050 served as the inclusion criterion and when *p* < 0.055 served as the exclusion criterion. HADS-D, Hospital Anxiety and Depression Scale, Depression Subscale; FSS, Fatigue Severity Scale; fVAS, fatigue Visual Analog Scale; SF36vs, Vitality subscale of the Medical Outcomes Study 36-Item Short-Form Health Survey; SF-36 pain, Pain subscale of the Medical Outcomes Study 36-Item Short-Form Health Survey; V12, Follow up visit after 12 months of a gluten-free diet. SF-36 pain scores were converted to facilitate comparisons, as follows: inverted SF-36 pain score = 100 − SF-36 pain score.

### Quality of life

After a one-year GFD, patients showed improved SF-36-questionnaire scores for Physical Functioning, General Health, Role-Physical, and Mental Health. Nevertheless, these scores remained lower than those observed in healthy control subjects (Table 3). The SF-36-questionnaire Role-Emotional scores were statistically different between V0 and V12, but the change was negligible. SF-36-questionnaire Social Functioning scores did not change in patients after the one-year GFD, and they were lower than those observed in healthy control subjects (Table 3).

**Table 3:** Depression, pain, and quality of life scores in patients with celiac disease, at diagnosis (V0), after 1 year of a gluten-free diet (V12), and in healthy control subjects.

	Patients at V0 (*n* = 78)	Patients at V12 (*n* = 78)	Change from V0 to V12 (95%CI)	value of *p* for V0 vs. V12	HCS (*n* = 78)	value of *p* for V12 vs. HCS
**Depression and pain scores**						
HADS-D (depression)	3.0 (1–7)	2 (1–4)	0.0(−1.0–0.0)	<0.001	1 (0–2)	<0.001
SF-36 pain (inverted)	44.0 (26–59)	26.0 (0–49)	−12.5 (−18.4−−6.6)	<0.001	10 (0–20.6)	<0.001
**Quality of life measures**						
**Physical health**						
Physical functioning	85 (75–95)	95 (85–100)	5.0 (0.0–5.0)	<0.001	100 (95–100)	0.006
General health	64.5 (47–82)	73.5 (57–88.3)	5.0 (0.0–7.5)	0.01	85 (77–97)	<0.001
Role-physical	50 (0–100)	100 (50–100)	25.0 (0.0–25.0)	<0.001	100 (100–100)	0.003
**Mental health**						
Social functioning	50 (50–50)	50 (50–50)	0.0 (0.0–0.0)	0.92	88 (76–92)	<0.001
Mental health	74 (60–80)	0 (71–88)	4.0 (0.0–8.0)	<0.001	88 (76–92)	0.004
Role-emotional	100 (33.3–100)	100 (66.7–100)	0.0 (0.0–0.0)	0.001	100 (100–100)	0.002

Data for V0, V12, and HCS are presented as the median (IQR). Data for the change from V0 to V12 are presented as the median (95% CI), except for the SF-36 Pain, which is presented as the mean (95% CI). Missing data: *n* = 2 for Mental Health, *n* = 1 for Role-Physical subscale scores in the control group. CI, Confidence interval; HADS-D, Hospital Anxiety and Depression Scale; Depression Subscale; HCS, Healthy Control Subjects; SF-36 pain, Pain subscale of the Medical Outcomes Study 36-Item Short-Form Health Survey; V0, First study visit; V12, Follow-up visit after 12 months of a gluten-free diet. SF-36 pain scores were converted to facilitate comparisons, as follows: inverted SF-36 pain score = 100 − SF-36 pain score.

### Persistent villous atrophy

Of 52 patients, 12 (23%) had small-intestine histologic findings that showed persistent villous atrophy (Marsh 3) after 1 year of GFD (V12). However, fatigue scores were not different between patients with Marsh 3 and those with Marsh 0/1, at V12 (Supplementary Table S2).

### Concomitant autoimmune disease

Among patients with CD, we compared those with and without concomitant autoimmune disease. We found that the fVAS, FSS, and inverted SF-36vs scores did not differ between these two groups at V0 or at V12 (Supplementary Table S3).

### Active celiac disease

Patients with CD were dichotomized into active CD (defined as anti-tTG-IgA ≥ 7 and Marsh score ≥ 3; *n* = 5) and well-treated CD (defined as anti-tTG-IgA < 7 and Marsh score = 0; *n* = 20). We found no difference in fatigue scores between these groups (Supplementary Table S4).

## Discussion

This study showed that fatigue declined considerably in patients with CD after 1 year of a GFD. However, fatigue remained higher than that observed in healthy subjects, and it was associated with depressive mood and pain across all three fatigue measures. We did not find any consistent association between fatigue and routine hematological or biochemical tests, anti-tTG-IgA levels, or persistent villous atrophy indicative of active CD.

Previously, fatigue in CD was rarely investigated in longitudinal studies. The present study was the first to apply multiple fatigue instruments and prospectively follow a large patient cohort for 1 year. Our present findings were consistent with those of two other small studies (*n* = 3 and *n* = 7 patients) (16, 17), but two other cross-sectional studies revealed no differences in fatigue scores between patients on a GFD and those on a gluten-containing diet (18, 19). The results from the present prospective controlled study were based on three validated, generic fatigue measures; thus, they provided an objective, valid description of this phenomenon.

Fatigue has been consistently associated with depression in autoimmune and inflammatory conditions; this was also the case in our study. Both depression and fatigue are important elements in the sickness behavior response. These elements arise through common pathways that involve IL-1 and other proinflammatory cytokines (31–33). Additionally, there are overlapping symptomatologies between fatigue and depression; thus, the wording used to assess fatigue and depression are similar. Consequently, the similar descriptions are likely to influence associations based on questionnaire results.

Pain was also associated with fatigue in all previous studies that examined both items. Pain induces the sickness behavior response, because it signals bodily damage, and consequently, it leads to fatigue (34). Recently, an association was found between fatigue and the *RTP4* gene, which encodes a Golgi chaperone that influences opioid pain receptor function. Thus, this molecular pathway might explain the relationship between pain and fatigue (35).

Notably, although fatigue severity consistently declined after the GFD, it did not reach the levels observed in healthy subjects. One explanation for this result could be a lack of adherence to the GFD. From other studies as much as 40% of patients report difficulty adhering to the GFD, which could have limited improvements in quality of life, depression, and anxiety (7, 36). The CD autoantibodies (anti-tTG-IgA, DGP) and the degree of villous atrophy could be considered surrogate measures of non-adherence. However, we found no associations between these measures and fatigue severity. Consequently, we could not conclude that a lack of adherence was an important cause of persistent fatigue in this cohort.

In this study, fatigue severity was not associated with CD activity (i.e., the grade of villous atrophy or levels of anti-tTG-IgA antibodies). This finding was consistent with findings in other diseases, which also lacked associations between fatigue and disease activity, when a generic fatigue measure was used (i.e., fatigue scoring instruments that do not contain elements of disease items) (37, 38). Interestingly, a recent study in patients with CD that exhibited histologically healed mucosa after a long-term GFD showed that disease was ongoing at the molecular level, and gene expression was different from that in healthy individuals (39). Those findings suggests that subclinical disease activity might be involved in generating fatigue by stimulating fatigue signaling pathways. Another possible factor could be that the mechanisms that downregulate inflammation and immunity might contribute to fatigue, as suggested in studies on other inflammatory diseases (40–43). In our study, a fraction of patients had shown evidence of disease activity (e.g., elevated anti-tTG-IgA and signs of villous atrophy) after the GFD. However, fatigue was not significantly higher in this subpopulation than in patients with well-treated CD that showed no sign of active disease. We also tested whether an autoimmune comorbidity might contribute to persistent fatigue, despite the GFD. Indeed, 21% of our patients with CD had one or more additional autoimmune diseases, which is in accordance with other studies (44). However, we found no difference in fatigue between patients with and those without concomitant autoimmune conditions, an observation possibly owing to the low number of patients with concomitant autoimmune conditions.

In our study, after a year of GFD, quality of life improved, except in the subscores for “Social functioning” and “Role-emotional.” However, despite symptom relief and improved physical well-being after the dietary intervention, all quality of life domains were lower than in healthy subjects, consistent with previous studies (45). This finding might be partly explained by the daily practical problems associated with adhering to a GFD (6). Of note, it was previously shown that a reduced quality of life was related to both adherence to a GFD and to the perceived degree of difficulty in adhering to a GFD (46).

This study had several limitations. We did not assess adherence to the GFD with a validated questionnaire, like the celiac dietary adherence test. We did not analyze gluten immunogenic peptides. Moreover, we did not use a patient-reported outcome measure specific for CD; however, there is no consensus on outcome measures for assessing CD in trials. Finally, the 12-month follow-up after initiating the GFD was limited; thus, the course of fatigue beyond this time period remains unknown.

In conclusion, we showed that fatigue was prevalent and severe in CD. It was considerably improved by a GFD, but the GFD did not completely normalize fatigue after 12 months. Persistent fatigue was not associated with proxies of disease activity, such as persistent villous atrophy or elevated anti-tTG-IgA, but it was consistently associated with depression and pain. There is a need for further studies regarding the biological mechanisms involved in fatigue, and the long term effect of a gluten-free diet.

## Data availability statement

The original contributions presented in the study are included in the article/Supplementary material, further inquiries can be directed to the corresponding author.

## Ethics statement

The studies involving humans were approved by Regional Ethics Committee (REC South-East Norway 2011/2631). The studies were conducted in accordance with the local legislation and institutional requirements. The participants provided their written informed consent to participate in this study.

## Author contributions

BS acquired funding for the study, curated the data, led the investigation and analysis, and drafted and revised the paper. TG and RO conceptualized and designed the study. TG was the lead project administrator, the lead supervisor, and drafted and revised the paper. RO was the supporting supervisor and drafted and revised the paper. AH curated the data, was the supporting project administrator, and revised the draft paper. JK supported the formal analysis, validated the statistical methodology, and revised the draft paper. LA developed the software used for data curation and revised the draft paper. IS curated the data and revised the draft paper. All authors contributed to the article and approved the submitted version.

## Funding

This study was supported by a grant from the Research Department of Stavanger University Hospital. The funding source had no role in study design, data collection or analysis, or in the preparation or approval of the manuscript.

## Conflict of interest

The authors declare that the research was conducted in the absence of any commercial or financial relationships that could be construed as a potential conflict of interest.

## Publisher’s note

All claims expressed in this article are solely those of the authors and do not necessarily represent those of their affiliated organizations, or those of the publisher, the editors and the reviewers. Any product that may be evaluated in this article, or claim that may be made by its manufacturer, is not guaranteed or endorsed by the publisher.

## Abbreviations

CD: Celiac disease
FSS: Fatigue Severity Scale
fVAS: Fatigue Visual Analog Scale
GFD: Gluten free diet
HADS-D: The Hospital Anxiety and Depression Scale, Depression Subscale
HCS: Healthy control subjects
SF-36: Medical Outcomes Study 36-Item Short-Form Health Survey
SF-36vs: Vitality subscale of the Medical Outcomes Study 36-Item Short-Form Health Survey
V0: First study visit
V12: Follow up visit after 12 months of gluten free diet
