# Supplementary material for: Less, but not gone—gluten-free diet effects on fatigue in celiac disease: a prospective controlled study
Source: Front Med (Lausanne). 2023 Sep 12;10:1242512. doi: 10.3389/fmed.2023.1242512 (PMC10520960; doi:10.3389/fmed.2023.1242512)
Supplement: Supplementary file 1 [file Table_1.docx]

**S1**: Univariable regression analysis of selected demographic and clinical variables in 78 celiac disease patients after one year on gluten free diet.

| **Variables** | fVAS | | FSS | | SF-36vs (inverted) | |
| --- | --- | --- | --- | --- | --- | --- |
|  | β | p-value | β | p-value | β | p-value |
| *Demographics* | | | | | | |
| Age | 0.006 | 0.956 | -0.004 | 0.973 | -0.218 | 0.228 |
| Sex | -0.134 | 0.244 | -0.093 | 0.417 | **-0.231** | **0.042** |
| BMI | 0.072 | 0.534 | 0.017 | 0.886 | 0.051 | 0.659 |
| *Nutritrional status* | | | | | | |
| Cobalamine | 0.117 | 0.323 | 0.146 | 0.216 | 0.057 | 0.633 |
| Ferritin | **-0.250** | **0.03** | **-0.198** | **<0.001** | **-0.290** | **0.012** |
| Folic acid | **0.315** | **0.007** | **0.296** | **0.011** | 0.198 | 0.093 |
| Hemoglobin | 0.009 | 0.941 | 0.032 | 0.784 | -0.02 | 0.861 |
| Vitamine D | 0.072 | 0.539 | 0.095 | 0.414 | 0.074 | 0.524 |
| *Disease activity* | | | | | | |
| Marsh-classification | 0.122 | 0.388 | 0.181 | 0.199 | 0.057 | 0.687 |
| Anti-tTG-IgA | -0.042 | 0.726 | -0.071 | 0.559 | 0.042 | 0.728 |
| DGP-AGA | 0.107 | 0.353 | 0.057 | 0.619 | 0.034 | 0.766 |
| Active celiac disease | -0.049 | 0.815 | 0,081 | 0.699 | 0.088 | 0.676 |
| *Other clinical factors* |  |  |  |  |  |  |
| Concomittant autoimmune disease | 0.042 | 0.714 | -0.022 | 0.851 | 0.036 | 0.754 |
| HADS-D-score | **0.696** | **<0.001** | **0.648** | **<0.001** | **0.642** | **<0.001** |
| SF-36 Pain (inverted) | **0.494** | **<0.001** | **0.544** | **<0.001** | **0.461** | **<0.001** |

Statistically significant values in bold.

Abbreviations: anti-tTG-IgA = anti-tissue transglutaminase-IgA antibodies, BMI: Body mass index, DGP-AGA: Deamidated gliadin peptide IgG antibodies, FSS: Fatigue Severity Scale, fVAS: fatigue Visual Analog Scale, HADS-D: The Hospital Anxiety and Depression Scale, Depression Subscale, SF-36 pain: Pain subscale of the Medical Outcomes Study 36-Item Short-Form Health Survey, SF-36vs: Vitality subscale of the Medical Outcomes Study 36-Item Short-Form Health Survey, V12: Follow up visit after 12 months of gluten free diet

**S2:** Fatigue scores in celiac disease patients with and without villous atrophy after one year of gluten free diet (V12):

| **Fatigue instrument** | **Patients with Marsh 0 and 1 at V12 (n=40)** | **p-value** | **Patients with Marsh 3 at V12 (n=12)** |
| --- | --- | --- | --- |
| FSS-score | 1.9 (1.5-3.3) | 0.122 | 2.6 (1.9-4.8) |
| fVAS | 15 (8.5-42.0) | 0.241 | 35 (14.0-48.0 |
| SF-36vs (inverted) | 35 (27.5-52.5) | 0.768 | 35 (30.0-58.8) |

Data are presented as median (IQR).

Abbreviations: FSS: Fatigue Severity Scale, fVAS: fatigue Visual Analog Scale, SF-36vs: Vitality subscale of the Medical Outcomes Study 36-Item Short-Form Health Survey, V0: First study visit, V12: Follow up visit after 12 months of gluten free diet

**S3:**Fatigue scores in celiac patients with and without other autoimmune disease.

| **Fatigue instrument** | **Patients with other autoimmune disease V0 (n=13)** | **Patients without other autoimmune disease V0 (n=65)** | **p-value**  **V0** | **Patients with other autoimmune disease V12 (n=16)** | **Patients without other autoimmune disease V12**  **(n=62)** | **p- value**  **V12** |
| --- | --- | --- | --- | --- | --- | --- |
| FSS-score | 3.0 (2.4-4.9) | 3.8 (2.2-4.7) | 0.564 | 1.85 (1.0 – 3.8) | 1.95 (1.6-3.4) | 0.691 |
| fVAS | 29.0 (23.0-52.0) | 46.0 (19.0-67.0) | 0.341 | 9.0 (7.0-61.0) | 17.0 (10.0-40.0) | 0.752 |
| SF-36vs (inverted) | 65.0 (35.0-70.0) | 65.0 (45.0-75.0) | 0.586 | 40.0 (27.5-50.0) | 32.5 (25.0-55.0) | 0.686 |

Data are presented as median (IQR).

Abbreviations: FSS: Fatigue Severity Scale, fVAS: fatigue Visual Analog Scale, SF-36vs: Vitality subscale of the Medical Outcomes Study 36-Item Short-Form Health Survey, V0: First study visit, V12: Follow up visit after 12 months of gluten free diet

**S4**: Fatigue scores in patients with active celiac disease versus well treated celiac disease after one year of gluten free diet (V12).

| **Fatigue instrument** | **Patients with well treated celiac disease (n=20)** | **p-value** | **Patients with active celiac disease (n=5)** |
| --- | --- | --- | --- |
| FSS-score | 2.3 (1.3-3.7) | 0.472 | 2.9 (2.0-4.7) |
| fVAS | 22 (7.0-51.0) | 0.946 | 24 (14.0-46.0) |
| SF-36vs (inverted) | 40 (25.0-52.5) | 0.838 | 30 (30,0-55.0) |

Data are presented as median (IQR). Abbreviations: FSS: Fatigue Severity Scale, fVAS: fatigue Visual Analog Scale, SF-36vs: Vitality subscale of the Medical Outcomes Study 36-Item Short-Form Health Survey, V12: Follow up visit after 12 months of gluten free diet

Active celiac disease defined as anti-tTG-IgA ≥7 and Marsh ≥3

Patients with well treated celiac disease defined as normal anti-tTG and normal histology (Marsh = 0).
